# Fh15 Blocks the Lipopolysaccharide-Induced Cytokine Storm While Modulating Peritoneal Macrophage Migration and CD38 Expression within Spleen Macrophages in a Mouse Model of Septic Shock

**DOI:** 10.1128/mSphere.00548-18

**Published:** 2018-12-19

**Authors:** Marcos J. Ramos-Benitez, Caleb Ruiz-Jimenez, Jose J. Rosado-Franco, Willy D. Ramos-Pérez, Loyda B. Mendez, Antonio Osuna, Ana M. Espino

**Affiliations:** a University of Puerto Rico, Medical Sciences Campus, Department of Microbiology, San Juan, Puerto Rico; b School of Science & Technology Universidad del Este, Carolina, Puerto Rico; c Instituto de Biotecnologia, Grupo de Bioquimica y Parasitología Molecular, Departamento de Parasitologia, Universidad de Granada, Granada, Spain; University at Buffalo

**Keywords:** CD38, *Fasciola hepatica*, cytokines, fatty acid binding protein, macrophages, septic shock

## Abstract

Sepsis caused by Gram-negative bacteria is the consequence of an unrestrained infection that continuously releases lipopolysaccharide (LPS) into the bloodstream, which triggers an uncontrolled systemic inflammatory response leading to multiorgan failure and death. After scrutinizing the immune modulation exerted by a recombinant Fasciola hepatica fatty acid binding protein termed Fh15, our group demonstrated that addition of Fh15 to murine macrophages 1 h prior to LPS stimulation significantly suppresses the expression of proinflammatory cytokines tumor necrosis factor alpha (TNF-α) and interleukin-1β (IL1-β). The present study aimed to demonstrate that Fh15 could exert a similar anti-inflammatory effect *in vivo* using a mouse model of septic shock. Among the novel findings reported in this article, (i) Fh15 suppressed numerous serum proinflammatory cytokines/chemokines when injected intraperitoneally 1 h after exposure of animals to lethal doses of LPS, (ii) concurrently, Fh15 increased the population of large peritoneal macrophages (LPMs) in the peritoneal cavity (PerC) of LPS-injected animals, and (iii) Fh15 downregulated the expression on spleen macrophages of CD38, a cell surface ectoenzyme with a critical role during inflammation. These findings present the first evidence that the recombinant parasitic antigen Fh15 is an excellent modulator of the PerC cell content and *in vivo* macrophage activation, endorsing Fh15’s potential as a drug candidate against sepsis-related inflammatory response.

**IMPORTANCE** Sepsis is a potentially life-threatening complication of an infection. Sepsis is mostly the consequence of systemic bacterial infections leading to exacerbated activation of immune cells by bacterial products, resulting in enhanced release of inflammatory mediators. Lipopolysaccharide (LPS), the major component of the outer membrane of Gram-negative bacteria, is a critical factor in the pathogenesis of sepsis, which is sensed by Toll-like receptor 4 (TLR4). The scientific community highly pursues the development of antagonists capable of blocking the cytokine storm by blocking TLR4. We report here that a recombinant molecule of 14.5 kDa belonging to the Fasciola hepatica fatty acid binding protein (Fh15) is capable of significantly suppressing the LPS-induced cytokine storm in a mouse model of septic shock when administered by the intraperitoneal route 1 h after a lethal LPS injection. These results suggest that Fh15 is an excellent candidate for drug development against endotoxemia.

## Abstract (precis)

Sepsis is a potentially life-threatening complication of an infection. Sepsis is mostly the consequence of systemic bacterial infections leading to exacerbated activation of immune cells by bacterial products, resulting in enhanced release of inflammatory mediators. Lipopolysaccharide (LPS), the major component of the outer membrane of Gram-negative bacteria, is a critical factor in the pathogenesis of sepsis, which is sensed by Toll-like receptor 4 (TLR4). The scientific community highly pursues the development of antagonists capable of blocking the cytokine storm by blocking TLR4. We report here that a recombinant molecule of 14.5 kDa belonging to the Fasciola hepatica fatty acid binding protein (Fh15) is capable of significantly suppressing the LPS-induced cytokine storm in a mouse model of septic shock when administered by the intraperitoneal route 1 h after a lethal LPS injection. These results suggest that Fh15 is an excellent candidate for drug development against endotoxemia.

## INTRODUCTION

Toll-like receptors (TLRs) are among the most-studied pattern recognition receptors (PRRs) in charge of sensing invading pathogens. TLR4 is expressed on a wide range of immune cells, which specifically recognize bacterial lipopolysaccharide (LPS). Activation of TLR4 leads to the synthesis of proinflammatory cytokines and chemokines with the ultimate goal of identifying and destroying the pathogen (1). However, activation of the innate immune system occasionally leads to host tissue collateral damage, resulting in organ dysfunction and death (2). In recent years, the development of an antagonist of TLR4 or downstream signaling pathways that inhibit the cytokine storm has emerged as a realistic therapeutic goal against sepsis (3).

Because of their central anti-inflammatory character, helminth infections have recently been considered a feasible therapeutic alternative against a number of inflammatory medical conditions such as sepsis, inflammatory bowel diseases, and multiple sclerosis, among others (4, 5). Fasciola hepatica, one of the most prevalent parasitic helminths worldwide, is a master of immunomodulation. Throughout the infection, the parasite suppresses the Th1 immune response while establishing a polarized Th2 environment that facilitates the parasite’s persistence in the host and the establishment of long-lasting chronic infections (6, 7). Infections with F. hepatica have been used to attenuate the clinical symptoms of murine autoimmune encephalomyelitis (8) and prevent the development of type 1 diabetes in a nonobese diabetic mouse model (9). However, the immunoregulation associated with *F. hepatica* infection lacks specificity and results in a compromised immune system unable to respond effectively to bystander infections (10, 11). Therefore, if parasite-derived components are to be considered a potential treatment option, proper identification and description of their mechanisms of action in mediating immune-suppressive function are essential.

In a previous study, we demonstrated that a 14.5-kDa recombinant protein belonging to the *F. hepatica* fatty acid binding protein (FABP) Fh15 significantly suppresses LPS-induced interleukin-1β (IL-1β) and tumor necrosis factor alpha (TNF-α) in murine bone marrow-derived macrophages (BMDMs) and THP1-Blue CD14 cells *in vitro* (12). The present study is the first to demonstrate the helminth antigen Fh15 is a potent anti-inflammatory agent capable of suppressing the cytokine storm while concurrently modulating the dynamic of macrophages in the peritoneal cavity and the activation status of spleen macrophages in a mouse model of septic shock.

## RESULTS AND DISCUSSION

### Fh15 significantly reduces serum cytokine/chemokine storms in a mouse model of septic shock.

**FIG 1:**
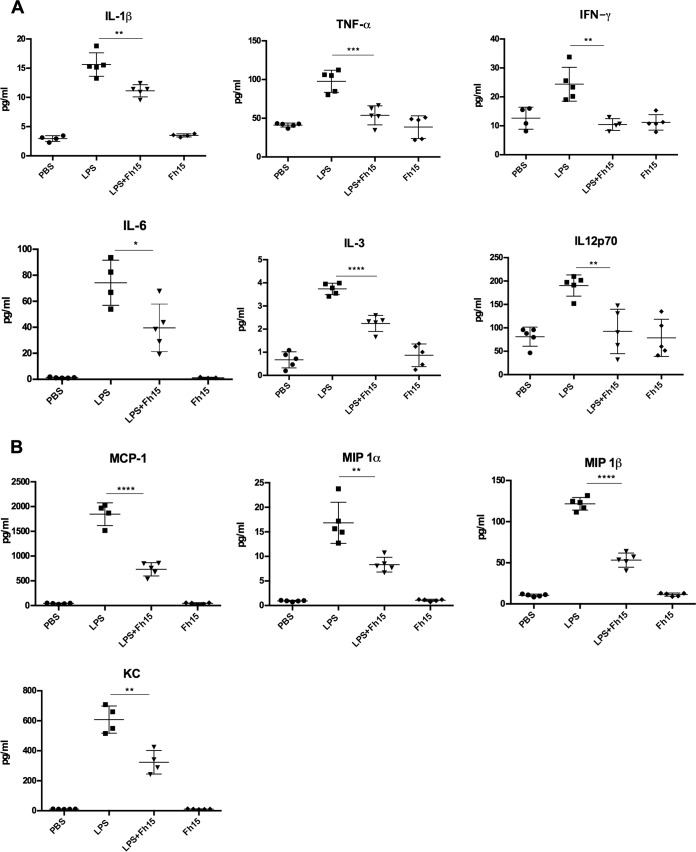
Fh15 suppresses inflammatory cytokines and chemokines *in vivo* in a murine model of sepsis. Groups of female 6- to 8-week-old BALB/c mice (*n* = 5) were injected i.p. with 50 μg Fh15 1 h after receiving a lethal i.p. injection with LPS (E. coli O111:B4 [7 mg/kg]). Control mice received PBS, Fh15, or LPS only (i.p.). Mice were sacrificed by cervical dislocation 12 h after LPS exposure, and blood samples were taken by orbital vein or cardiac puncture. A Bioplex mouse cytokine assay was used to measure the concentrations of cytokines (A) and chemokines (B) in serum.

**FIG 2:**
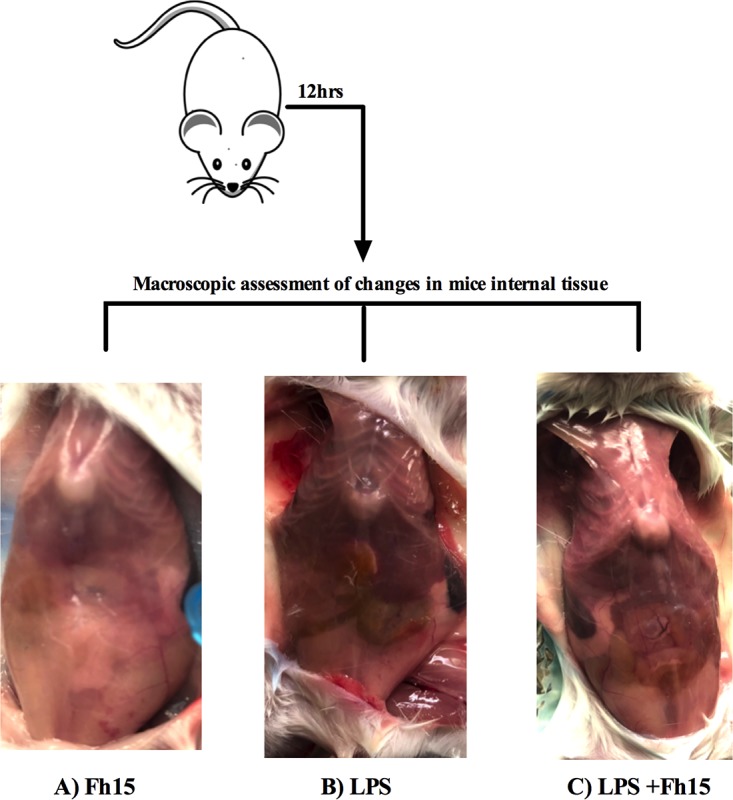
Macroscopic assessment of internal tissues. BALB/c mice injected (i.p.) with Fh15 (50 μg), LPS (E. coli O111:B4 [7 mg/kg]), or Fh15 1 h after LPS injection were sacrificed 12 h after the LPS injection and necropsied to examine the gross appearance of the peritoneal cavity (PerC). Pictures show that the PerC of animals treated with Fh15 exhibits a healthy appearance compared to that of LPS-treated animals.

Since we previously demonstrated that Fh15 significantly suppresses the expression of TNF-α and IL-1β from BMDMs and the LPS-induced NF-κB activation within THP1-Blue CD14 cells when it is added to culture up to 6 h after LPS stimulation (12), in the present study, we wanted to assess whether Fh15 could exert a similar anti-inflammatory effect *in vivo*. For this purpose, we developed a mouse model of septic shock using BALB/c mice, which is a prototypical Th2-type mouse strain that in the presence of LPS or live bacteria shows a relative impaired bactericidal activity (13), and consequently BALB/c is considered a mouse strain highly susceptible mouse to endotoxemia (3). Animals received a single intraperitoneal (i.p.) injection with a lethal dose of LPS (7 mg/kg body weight) (14) followed by a single i.p. injection of 50 μg Fh15 (1 h apart) and 12 h after the LPS insult were bled out and euthanized. As expected, animals that received only the LPS injection had significantly higher serum levels of proinflammatory cytokines than did animals that received only Fh15 or phosphate-buffered saline (PBS). Fh15 treatment significantly inhibited the LPS-induced serum levels of IL-1β (*P* < 0.0021), TNF-α (*P* < 0.0079), gamma interferon (IFN-γ; *P* < 0.0028), IL-12p70 (*P* < 0.0007), IL-6 (*P* < 0.0001), and IL-3 (*P* < 0.0233) (Fig. 1A). TNF-α, IL-1β, and IL-6 are major cytokines that act as endogenous pyrogens that regulate early responses during sepsis. These cytokines upregulate the synthesis of secondary mediators and other proinflammatory cytokines by macrophages and endothelial cells, stimulate the production of acute-phase proteins, and attract other leukocytes to the site with the purpose of control and infection clearance (15). IFN-γ, IL-3, and IL12p70 are important cytokines involved in macrophage activation (16, 17) and the differentiation of naive T cells into Th1 cells, respectively, and have been found increased in serum from septic patients (18). The observation that Fh15 simultaneously suppressed all these cytokines in animals exposed to a lethal dose of LPS is a clear indication that this molecule exerts a powerful role in minimizing sepsis pathogenesis progression. Consistent with these results, Fh15 also significantly suppressed the levels of a number of serum chemokines, such as monocyte chemoattractant protein 1 (MCP-1; *P* < 0.0001), macrophage inflammatory protein 1α (MIP-1α; *P* < 0.0078), and KC (*P* < 0.0033) (Fig. 1B), which have been examined in sepsis scenarios due to their important role in the recruitment of leukocytes to inflammation sites (19–22). In agreement with the suppression of cytokines and chemokines, the abdominal cavity of animals treated with Fh15 showed a gross macroscopic healthy appearance that was very similar to those observed within animals injected with PBS or Fh15 alone (Fig. 2). In contrast, the abdominal cavity from animals that only received the lethal LPS injection showed evident signs of hemorrhage, which is a clear indication of an ongoing inflammation leading to septic shock. These results are consistent with those previously reported for our research group using Fh12, the native form of *F. hepatica* FABP within C57BL6 mice, a prototypical Th1-type mouse strain with highly bactericidal capacity and consequently much more resistant to endotoxemia (23), which also showed significant suppression of the cytokine storm and sepsis progression. Collectively, these results confirm the potent anti-inflammatory effect of *F. hepatica* FABPs and their role as TLR4 antagonist and suggest that Fh15 could be used to therapeutically block the adverse biological consequences of endotoxemia *in vivo* therapeutically.

### Fh15 promotes persistence of large peritoneal macrophages in the peritoneal cavity.

**FIG 3:**
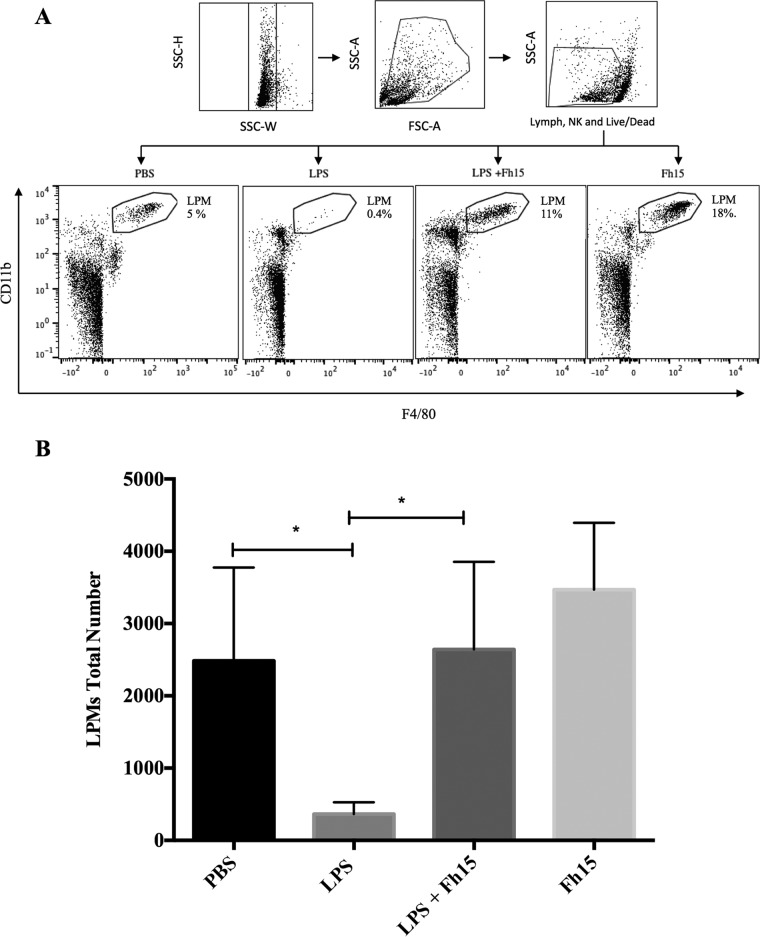
Fh15 promotes persistence of large peritoneal macrophages in the peritoneal cavity. Peritoneal exudate cells (PECs) were collected from BALB/c mice (6 to 8 weeks old) injected i.p. with 50 μg Fh15 1 h after LPS injection (E. coli O111:B4 [7 mg/kg]), LPS alone, Fh15 alone, or PBS. Cells collected from animals of the same experimental group (*n* = 5) were pooled and labeled with a cocktail of specific antibodies against CD4^+^, CD8^+^, B220^+^, and NK1.1^+^ to exclude all these cells, including dead cells, by gating. LPMs were identified via their high levels of expression of F4/80 and CD11b. The percentage at the upper right part of the figure represents the amount of LPM relative totals to the cells at the PerC after each treatment. (A) We observed a substantial reduction in the number of LPMs within the LPS-injected group. In contrast, animals treated with Fh15 had a more prevalent LPM population. The data shown are a representative example from an independent experiment. (B) Comparison of the total number of LPMs gated after the different treatments. The data shown represent the average ± SD from three independent experiments. Flow cytometry data were acquired on a Miltenyi MACSQuant Analyzer 10 instrument. Data were analyzed with FlowJo software (FlowJo, LLC).

There are a significant number of peritoneal macrophages in mice, which play a role in the clearance of endotoxin (24) and coordinating inflammatory responses (25, 26). Recently, the coexistence of two different macrophage subsets in the mouse peritoneal cavity (PerC) was reported, which have been classified according to their morphology as small peritoneal macrophages (SPMs) and large peritoneal macrophages (LPMs) (27). LPMs are the most abundant subset under steady-state conditions and are characterized by expression of high levels of F4/80 and CD11b and low levels of major histocompatibility complex class II (MHC-II). SPMs, a minor subset in unstimulated PerCs, express lower levels of F4/80 and CD11b and high levels of MHC-II (27). Given the potent suppressive effect that Fh15 showed against the production of proinflammatory cytokines in septic mice, we wanted to investigate whether Fh15 could exert any influence on the dynamics of LPM and SPM subsets present within the PerC of animals exposed to LPS. Using PBS, we washed the PerC of mice injected with LPS, Fh15, or LPS before the Fh15 treatment and labeled cells with specific anti-CD11b and F4/80 antibodies. Our gating strategy excluded dead cells, lymphocytes, and NK cells. Next, we separated the SPM and LPM populations based on their differential expression of F4/80 and CD11b markers (27). In agreement with previous reports (27), the LPM population in the PerC of mice injected only with LPS was significantly reduced (*P* = 0.0475) compared to animals injected with PBS (Fig. 3A). The disappearance of LPMs from PerC after inflammatory stimuli has been associated with the migration of these cells to the omentum (28), which provides the correct microenvironment and growth factors for macrophage proliferation and maturation (29, 30). LPMs return to PerC several days after that to resolve the inflammatory process (31). Concurrently, the SPM subset becomes the predominant macrophage population, likely due to a large number of blood monocytes rapidly entering the PerC and differentiating to SPMs (27). The results demonstrate that the treatment with Fh15 1 h after LPS insult shifted this dynamic. The LPM population did not disappear and became significantly more abundant than the LPS-injected animals (*P* = 0.0394) (Fig. 3B). The high abundance of LPMs in the Fh15-treated animals is also consistent with the observation that although Fh15 decreases the LPS-induced levels of chemokines, the levels of chemokines in serum remained higher than that in PBS-treated animals (Fig. 1B). This event suggests chemotaxis of other cells besides macrophages, such as neutrophils, mast cells, and lymphocytes to the peritoneal cavity. Since our gating strategy excluded cell populations other than macrophages, we were unable to determine if and to what extent these other cells could have contributed to the remaining chemokine levels observed in the Fh15-treated animals.

**FIG 4:**
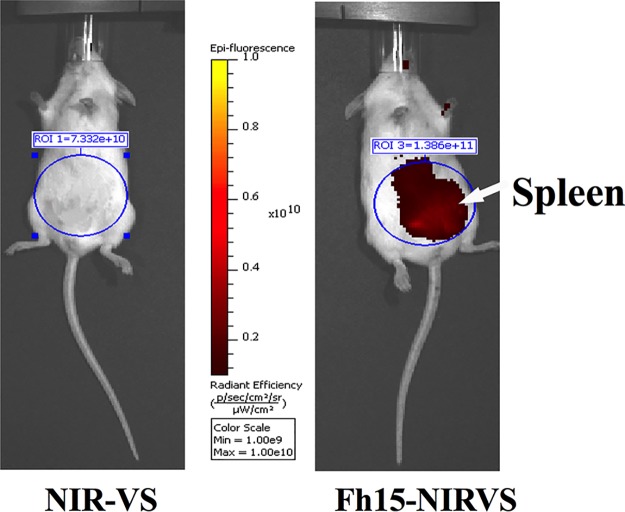
Fh15 remains in large detectable amounts in the peritoneal cavity of mice. BALB/c mice were i.p. injected with Fh15 (50 μg) conjugated to NIR-VS (Fh15–NIR-VS) (*n* = 2) or NIR-VS–lysine-conjugated molecule (*n* = 2). Animals were anesthetized, and the Fh15 distribution was mapped at different time points (30 min and 2 h, 12, and 24 h) after injection using an IVIS Lumina-II (Caliper LifeScience). No significant signal was present in the NIR-VS control mice. In contrast, a strong fluorescence signal from Fh15–NIR-VS was measured in the injected mouse, indicating that Fh15 remained at high concentrations throughout the PerC with a tendency to localize toward the spleen. The image represents the localization of Fh15 24 h after injection.

The effect caused by Fh15 on the dynamic of macrophage populations is consistent with the effect that *F. hepatica* newly excysted juveniles (NEJs) cause during their migration through the PerC. Immediately after excystment, NEJs cross the intestinal wall and migrate into the PerC for approximately 3 days after infection (32). During this phase, the parasite recruits a large number of immune cells, specifically macrophages, which are alternatively activated (M2-type macrophages) (33, 34). It is thought that the control of the host immune system by *F. hepatica* likely begins at this moment. This description is consistent with our observation that Fh15 labeled with a fluorescent organic compound, NIR-783-piperazima-vinyl sulfone (Fh15–NIR-VS), remains at relatively high abundance throughout the PerC for 24 h, with a tendency to localize toward the spleen, as evidenced by IVIS-Lumina image (Fig. 4). The observation that Fh15 can promote the persistence of the LPM population within the PerC leads us to suggest a primary modulatory mechanism based on the maintenance of a prolonged steady state in the peritoneum even in the presence of an inflammatory stimulus.

### Fh15 downregulates overexpression of CD38 provoked by LPS insult on spleen macrophages.

**FIG 5:**
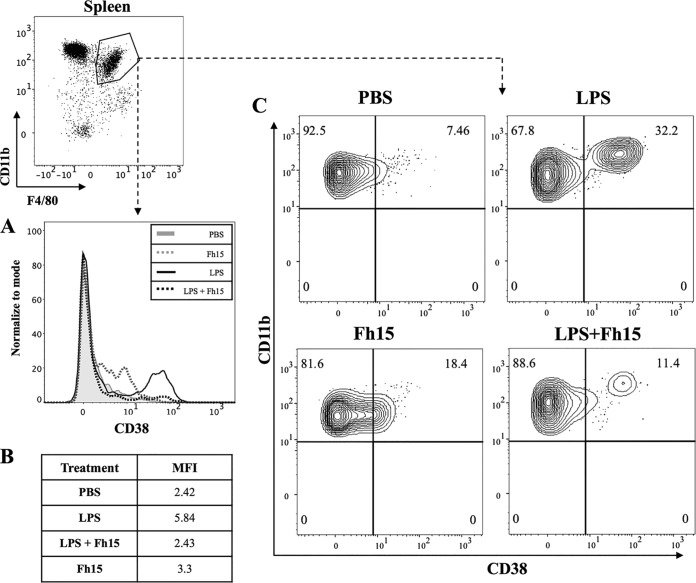
Fh15 downregulates overexpression of CD38 provoked by LPS insult on the spleen macrophage population. Groups of mice were divided and treated as previously described. After gating, CD38 expression was measured in macrophages (CD11b^+^ F4/80^+^) from spleen. (A) Flow cytometry histogram showing a decrease in CD38 after Fh15 exposure 1 h post-LPS stimulation. (B) Comparison of CD38 expression by mean fluorescent intensity (MFI) values among treatments. (C) Alternative contour plots also showing Fh15’s effect on CD38 expression in LPS-treated mice.

Recently, it was shown that CD38 constitutes a novel marker delineating M1 macrophage differentiation after LPS exposure in mice (35). CD38 participates in the NAD hydrolysis and NAD-dependent synthesis of cyclic ADP-ribose (cADPR) and also functions in cell adhesion, signal transduction, and calcium signaling (36). We aimed to determine whether Fh15 could exert any effect on the expression of this marker within spleen macrophages of animals that received the treatment with Fh15 after the lethal LPS injection. To assess this, we labeled cells with specific antibodies for CD11b^+^, F4/80^+^, and CD38. Spleen macrophages from animals only exposed to LPS showed an increase in CD38 expression compared to that of animals exposed to PBS or Fh15 alone. In the presence of Fh15, the LPS-induced expression of CD38 was decreased (Fig. 5), which suggests that Fh15 could prevent the LPS-induced M1-type macrophages.

Keeping in mind that CD38 participates in the cADPR synthesis regulating intracellular calcium (Ca^2+^) in many types of cells (37), that Ca^2+^ levels are associated with an effective innate immune response, specifically through TLR4, that LPS induces Ca^2+^ entry in endothelial cells in a TLR4-dependent manner, and that disruption of Ca^2+^ entry to cells renders protection against sepsis-induced lethality (38), we could speculate that the blockage of the CD38/cADPR pathway in animals exposed to LPS would reduce intracellular Ca^2+^ in a similar manner, as has been observed to occur in microglia stimulated with LPS *in vitro* (39). In that regard, the observation that Fh15 affects the expression of CD38 within spleen macrophages from animals exposed to LPS, which had significantly suppressed the levels of serum proinflammatory cytokines/chemokines, could suggest a possible immune modulation mechanism for Fh15 based on the interference of the releasing/influx levels of Ca^2+^ from the cell. However, we understand that at this point, these are assumptions, and more work directed to address this hypothesis is being scheduled.

In summary, this is the first study to report the anti-inflammatory capacity of recombinant *F. hepatica* fatty acid binding protein (Fh15) *in vivo* using a mouse model of septic shock. Although not necessarily linked in their mechanism, this work presents three novel contributions to the developing field of helminth molecules with anti-inflammatory properties: (i) Fh15 suppressed the production of inflammatory mediators *in vivo* after inflammation onset, (ii) Fh15 prevented the “disappearance” of LPMs from the PerC, counterbalancing the macrophage populations within the PerC during inflammation and promoting a persistent state and homeostasis in the peritoneum after the LPS insult, and (iii) Fh15 suppressed the expression of CD38 levels of spleen macrophages, which could indicate a possible role for Fh15 in the suppression of M1-type macrophage polarization characteristic of hyperinflammation caused by LPS.

## MATERIALS AND METHODS

### Animals.

Female wild-type BALB/c mice 4 to 6 weeks old (Charles River) were bred and kept under pathogen-free conditions. Upon arrival, mice were housed five animals per cage with free access to food and water. All animal experimental procedures were approved under University of Puerto Rico Medical Science Campus IACUC protocol no. 7870215, which follows AAALAC guidelines.

### Production of recombinant Fh15.

cDNA expressing Fh15 was cloned in the pGEX-4T-2 expression vector (40), and the construct was propagated and expressed in Escherichia coli TOP10. High-throughput protein expression, scale-up, and purification were performed exactly as previously described (12). Batches of Fh15 were tested using a commercially available Chromogenic Limulus assay (Pierce, USA) as previously described (12). Protein concentration was determined by the bicinchoninic acid (BCA) method (Pierce, Rockford, IL).

### Synthesis of NIR-783-VS, coupling to Fh15, and tracing of Fh15–NIR-VS in mice.

**FIG 6:**
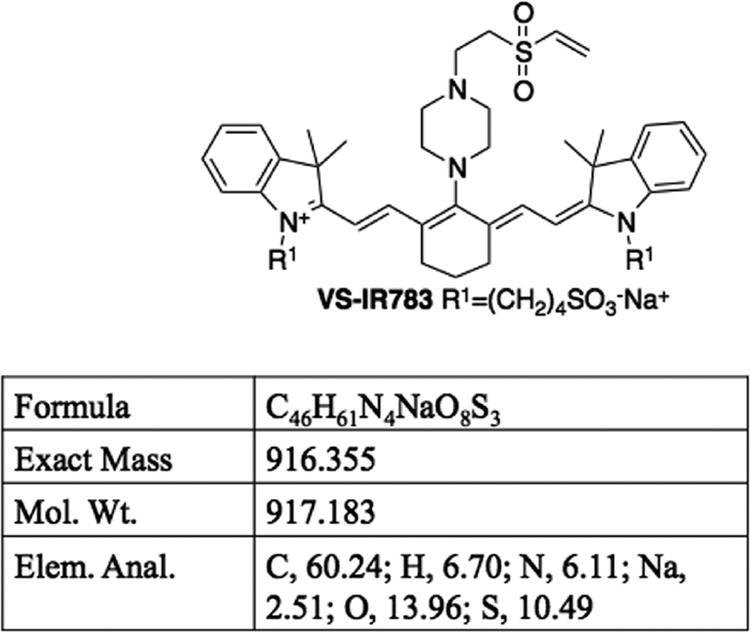
Structure and characteristics of NIR-783-piperazima-vinyl sulfone (NIR-VS). NIR-VS is a synthetic organic compound of 917,183 Da that possesses the property to excite and emit light at 490 and 504 nm, respectively. Fh15 was conjugated to the sulfone groups (S = O) of this compound via free amino groups. This compound was synthesized and kindly donated by the Institute of Biotechnology of the University of Granada, Spain.

NIR-783-piperazima-vinyl sulfone (NIR-VS) (Fig. 6) is a synthetic organic compound of 917,183 Da that possesses the property to excite and emit light at 490 and 504 nm, respectively. This product is a derivate from one previously used in immunization studies (41) and was kindly donated by the Institute of Biotechnology of University of Granada, Spain. NIR-VS was dissolved in PBS at a final concentration of 2 mg/ml, mixed with the purified Fh15 (2 mg/ml) in PBS, and kept overnight at 4°C in an orbital shaker. Free reactive groups were blocked with a molar excess of glycine in carbonate buffer at room temperature for 4 h. Two mice were injected (i.p.) with 50 μg of Fh15–NIR-VS or NIR-VS alone. Animals were anesthetized (ketamine/xylazine), and the presence of Fh15 was traced *in vivo* at 30 min and 2, 12, and 24 h after injection using an IVIS Lumina-II (Caliper LifeScience).

### Septic shock induction and serum cytokine/chemokine-level measurement.

Groups of 5 animals each were injected (i.p.) with 7 mg/kg of body weight LPS (E. coli O111:B4 [Sigma-Aldrich]), and 1 h thereafter, animals received a single i.p. injection of 50 μg Fh15 in PBS. Control mice were injected with PBS, Fh15, or LPS only (i.p.). Mice were sacrificed by cervical dislocation 12 h after the last injection and necropsied for collection of peritoneal exudate cells (PECs) and spleen. Animals were bled from the orbital vein or by cardiac puncture. Serum concentrations of cytokines (TNF-α, IL-1β, IL-6, IL-12p70, IL-3, and IFN-γ,) and chemokines (MCP-1, KC, MIP-1a, and MIP-1b) were measured by using a Bioplex mouse cytokine assay (Bio-Rad, Hercules, CA). Additionally, a gross macroscopic analysis was performed to assess differences between groups in pathological appearance of the peritoneal cavity.

### PECs and flow cytometry analysis.

PECs were harvested by washing the PerC with 10 ml of cold PBS as described elsewhere (42). Spleens were excised into small fragments and then pressed through a strainer using the plunger end of a syringe and washed several times with PBS. Next, the suspension was washed several times by hypotonic lysis to remove erythrocytes and then resuspended in PBS by vigorous pipetting as previously described (43). Splenocytes or PECs were washed twice with PBS containing 2% fetal bovine serum (FBS) and adjusted to 1 × 10^6^ cells/ml. Cells were stained with different antibodies to identify macrophages. First, CD4^+^, CD8^+^, B220^+^, NK1.1^+^, and dead cells were excluded by gating. Macrophages were identified as F4/80^+^ CD11b^+^ cells. The following antibodies were used in these experiments: anti-F4/80-fluorescein isothiocyanate (FITC) (BM8), CD11b-phycoerythrin (PE)/Cy7 (M1/70), CD38-PE (90), CD4-PacBlue (GK1.5), CD8-PacBlue (53-6.7) NK-1.1–PacBlue (PK-136), and CD45R-PacBlue (RA3-62B) (Biolegend, San Diego CA). Cells collected from animals of the same experimental group were pooled, washed twice with PBS containing 2% FBS, and fixed with 1% paraformaldehyde. Cell populations were analyzed on a Miltenyi MACSQuant Analyzer 10 instrument. Data were analyzed with FlowJo software (FlowJo, LLC). To distinguish autofluorescing cells from cells expressing low levels of individual surface markers, we established upper thresholds for autofluorescence by staining samples with fluorescence-minus-one control stain sets (44, 45), in which a reagent for a channel of interest is omitted.

### Statistical analysis.

The experiments were repeated three times on different days, and each independent experiment included three replicates. Data were expressed as mean values ± standard deviations (SD) for each determination. Statistical significance was determined by Student's *t* test for single comparisons or one-way analysis of variance (ANOVA) for multiple comparisons using GraphPad Prism software (Prism-6). For all tests, a *P* value of <0.05 was considered significant.
